# Adopting Consensus Terms for Testing in Precision Medicine

**DOI:** 10.1200/PO.21.00027

**Published:** 2021-10-06

**Authors:** Nikki A. Martin, Joel E. Tepper, Veda N. Giri, Thomas E. Stinchcombe, Heather H. Cheng, Milind M. Javle, Eric Q. Konnick

**Affiliations:** ^1^ LUNGevity Foundation, Bethesda, MD; ^2^ Department of Radiation Oncology, UNC/Lineberger Comprehensive Cancer Center, UNC School of Medicine, Chapel Hill, NC; ^3^ Cancer Risk Assessment and Clinical Cancer Genetics, Departments of Medical Oncology, Cancer Biology, and Urology, Sidney Kimmel Cancer Center, Thomas Jefferson University, Philadelphia, PA; ^4^ Duke Cancer Institute, Duke University Medical Center, Durham, NC; ^5^ Clinical Research Division, Fred Hutchinson Cancer Research Center, Seattle, WA; ^6^ Department of Medicine, Division of Oncology, University of Washington, Seattle, WA; ^7^ Seattle Cancer Care Alliance, Seattle, WA; ^8^ Department of Gastrointestinal Medical Oncology, The University of Texas MD Anderson Cancer Center, Houston, TX; ^9^ Department of Laboratory Medicine and Pathology, University of Washington, Seattle, WA

## Abstract

Despite the well-understood benefits of biomarker and genetic testing in precision medicine, uptake remains low, particularly for patients with low socioeconomic status and minority ethnic backgrounds. Patients report having limited familiarity with testing terminology and may not be able to accurately explain testing's role in treatment decisions. Patient confusion and lack of understanding is exacerbated by a multiplicity of overlapping terms used in communicating about testing. A LUNGevity Foundation–led working group composed of five professional societies, 23 patient advocacy groups, and 19 industry members assessed and recommended specific terms for communicating with patients on testing for tumor characteristics and germline mutations.

**METHODS:**

Members completed a precision oncology testing framework analysis (biomarkers, germline variants, testing modalities, biospecimen, and commonly used testing terms) for nine solid tumors and blood cancers. The evaluation was segmented into terms that distinguish between somatic and germline testing. Additional data were captured in a comprehensive survey (1,650 respondents) led by FORCE (Facing Our Risk of Cancer Empowered) on patient preferences on germline testing terms.

**RESULTS:**

Thirty-three terms were noted in patient education related to biomarker, genetic, and genomic testing. Biomarker testing was selected as the preferred term for testing for somatic (acquired) alterations and other biomarkers. Genetic testing for an inherited mutation and genetic testing for inherited cancer risk were selected as the preferred terms for testing for germline variants.

**CONCLUSION:**

Democratizing comprehension about precision oncology testing through intentional use of plain language and common umbrella terminology by oncology health care providers and others in the oncology ecosystem may help improve understanding and communication, and facilitate shared decision making about the role of appropriate testing in treatment decisions and other aspects of oncology care.

Precision medicine has transformed the practice of oncology, offering opportunities for significantly improved outcomes in an array of solid and hematologic malignancies. Indeed, professional guidelines routinely recommend the application of genomic and laboratory techniques in oncology to both direct treatment and elucidate inherited cancer risks.

**CONTEXT**

**Key Objective**
Advanced diagnostic cancer risk and oncology testing that informs personalized treatment decisions for patients has been challenging to communicate effectively because of medical jargon and overlapping terminology. For the first time, a multistakeholder pan-cancer working group analyzed the landscape of precision-medicine terminology and provided recommendations for plain language terms that providers and other stakeholders can use to address gaps in patient health literacy and improve shared decision making.
**Knowledge Generated**
Recommended consensus umbrella testing terms for patient communication were biomarker testing (for acquired tumor characteristics) and genetic testing for an inherited mutation and genetic testing for inherited cancer risk (for germline testing).
**Relevance**
A recently updated CDC definition of health literacy incorporates the role of organizations in making health information equitably accessible and understandable to patients. When used consistently, common cancer testing terminology can address poor patient comprehension of the role of testing in accurate treatment selection.

However, many eligible patients are not benefiting from advances in precision medicine because of low rates in both biomarker testing for tumor-specific therapies and genetic testing for inherited mutations that indicate increased cancer risk. In lung cancer, for example, a study of 5,688 patients with non–small-cell lung cancer from 2011 to 2016 demonstrated that 15.4% received broad-based genomic sequencing and 84.6% received single gene testing for *EGFR* and/or *ALK*.^1^ A more recent study evaluating testing rates showed that only 7% of patients receiving care in community oncology settings received the recommended testing for all seven biomarkers specified in the active clinical guidelines.^2^ Likewise, in patients with gastrointestinal stromal tumor, recent data indicate that fewer than 27% received recommended tumor testing for *KIT* mutations,^3^ and only 40% of patients with colorectal cancer received recommended testing for known actionable mutations.^4^ Testing according to current guidelines remains below 50% for most populations recommended for inherited cancer risk testing. This includes subgroups of patients with breast cancer, and patients with ovarian, pancreatic, and metastatic prostate cancer.^5^

There are multiple likely reasons for this pervasive undertesting, including limited availability of adequate samples, lack of provider knowledge or support (including testing and counseling resources), geographic factors, racial disparities, socioeconomic factors, limited insurance coverage, and reimbursement challenges.^6^ Studies suggest that demographic factors such as language, age, and insurance status may contribute to decreased access to germline genetic testing in prostate cancer.^7^ In colorectal cancer, study findings suggest that socioeconomic status, insurance status, and hospital care settings could also play a role in access to biomarker testing.^8^ Recent studies have shown lower biomarker testing rates in patients with cancer from underserved communities.^9,10^ For example, a recent retrospective observational study of patients with non–small-cell lung cancer using the Flatiron Health database showed a more than 10 percentage point difference in White (50.1%) patients receiving biomarker testing with next-generation sequencing compared with Black patients (39.8%).^11^ Inequitable access to testing and treatment is also exacerbated by inadequate inclusion of diverse ethnicities in the diagnostic test reference cohorts compared with populations of these patients receiving testing in the clinic. One study showed that the proportion of American Indian or Alaskan Native and Native Hawaiian or Other Pacific Islander in a pan-cancer institutional cohort receiving NGS testing was significantly lower than that of patients of European ancestry.^10^

Managing these complex challenges will require long-term policy, process, and infrastructure solutions, but there are also more immediate opportunities at hand to address a key driver of suboptimal testing rates: confusion and lack of understanding among patients and caregivers about the language used in precision medicine. Recent preliminary results (manuscript in preparation) from Cancer Support Community, a pan-cancer patient advocacy organization, found wide variability of familiarity of terms used in patient education about precision medicine. The research surveyed 30 patients and caregivers (21 women and nine men) with education levels ranging from high school to post-graduate school who have experience with malignancies such as breast cancer (eight), prostate cancer (six), lung cancer (three), and other cancers (13). Almost two thirds of patients with cancer and caregivers reported never having heard of or not knowing anything about the terms precision medicine (61%) and cancer subtype (66%). When asked about biomarker testing, which can also be called molecular testing, tumor profiling, somatic testing, or genomic testing of cancer cells, most patients and caregivers reported being familiar with the term and were able to articulate an accurate definition. However, although 73% indicated a basic understanding of targeted therapy, most respondents were unable to provide an accurate definition. Most respondents (90%) reported they understood the term genetic testing for inherited cancer risk and were able to articulate an accurate meaning (Table 1).

**TABLE 1.**
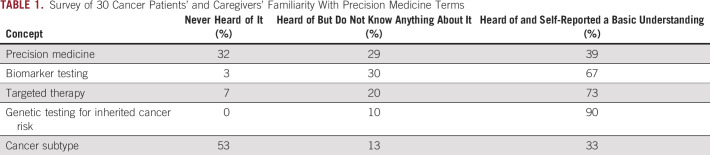
Survey of 30 Cancer Patients' and Caregivers' Familiarity With Precision Medicine Terms

Additional data from a separate study (manuscript in preparation) by the patient advocacy group LUNGevity Foundation that surveyed patients with lung cancer about recognition of precision medicine terms found that awareness of the term targeted therapy has penetrated the patient population. When patients with lung cancer were asked whether they had heard the terms biomarker testing, mutation testing, genetic testing, genomic testing, tumor profiling, and molecular testing, 88% of patients responded that they had heard of at least one testing term. The somatic mutation testing term that had the highest level of familiarity to patients with lung cancer was biomarker testing, with 92% of patients in the LUNGevity network and 65% of patients who are unaffiliated with a patient advocacy group citing familiarity.

Patient-reported confusion about biomarker testing is likely driven, in part, by the lack of consistency and multitude of different terms used by providers, other experts, and commercial companies when discussing testing.^12^ When asked, patients often cite difficulty sorting out complex information about what tests to have, what tests they may have had, how results can guide treatment and care decisions, and how test results may apply to patients accessing clinical trials. In a survey of 648 patients with breast cancer from diverse communities conducted by patient advocacy groups Facing Our Risk of Cancer Empowered (FORCE) and Living Beyond Breast Cancer, respondents seemed to understand germline genetic testing the most, and tumor biomarker testing for acquired alterations the least. Almost half of the respondents (46%) reported that they did not understand their tumor biomarker test results. In addition, some respondents expressed confusion about the difference between genetic testing for inherited mutations (pathogenic variants) and tumor testing for acquired mutations only found in the tumor.^13^

## PROPOSAL

Although precision medicine and testing can be complicated subjects for a lay audience, it is important that the medical community and others communicating with patients strive for language that is both accurate and accessible so that our patients can be active partners in managing their care and engaging in shared decision making about their best care options.

To assess the extent of patient and caregiver confusion about testing terminology and propose potential remedies, over the last year, LUNGevity Foundation has convened a multistakeholder pan-cancer working group of patient advocacy organizations, professional societies, medical product developers, and laboratories.

After documenting more than 33 different terms related to biomarker, genetic, and genomic testing that are currently in use within oncology clinical care and patient education, the working group sought consensus among the stakeholders on several proposed preferred terms that could be applicable across tumor types.^14^ These terms were evaluated by working group members, which included patient advocacy organizations with expertise in precision medicine for their disease space, professional societies such as the Association for Molecular Pathology, the Association of Community Cancer Centers, and the National Society of Genetic Counselors, and industry represented by pharma and biotech, laboratories, and test manufacturers. In addition, for the selection of the germline testing term, terms were refined based on feedback received through surveys of more than 1,700 patients and caregivers. Although working group members did not perform an additional patient survey for the selection of a term for testing for acquired somatic and nongenomic biomarkers, a subset of member organizations had previously queried their patient communities about terms under consideration, and these insights were integrated into the discussion and selection of a preferred term.

The result of this effort was the recommendation that all stakeholders in the oncology ecosystem adopt common, consistent terms for biomarker and germline genetic testing for all cancer types. Specifically, the working group proposed the following:

1. For tests that identify characteristics, targetable findings, or other test results originating from malignant tissue or blood, the recommended umbrella term is *biomarker testing.*
2. For tests that identify germline mutations or variants, the recommendation is for *genetic testing for an inherited mutation* and *genetic testing for inherited cancer risk*, which would be used in the appropriate specific clinical scenario.

These recommendations, which are detailed in a recently released White Paper,^15^ are designed to be cross-cutting umbrella terms that can be used in all care settings, with the recognition that there will be important nuances relating to individual patients' specific disease states and family histories. It is expected that providers and others who communicate with patients will augment the baseline terms with necessary additional explanations, to ensure that patients receive accurate and appropriate information about their diagnosis, prognosis, and care options. It should be noted that providers who practice in diverse cross-cultural and multiracial communities where English is not the first language may benefit from additional adaptation of these terms for optimal provider-patient communication. Guiding principles for cultural adaptation include a four-step process: (1) forward translation; (2) expert panel review of the translated terms; (3) back-translation, and (4) testing the terms with the intended audience in interviews and focus groups.^16^

Adopting a consistent set of clear, plain-language terms as the starting point for improved patient and provider communication and understanding is a critical step in maximizing the potential benefit of novel therapeutic approaches for our patients. We applaud and support the working group's commitment to this goal and encourage our oncology provider colleagues to join us in adopting these recommendations.
